# Quadricuspid Aortic Valve: An Incidental Finding in an Elderly Man

**DOI:** 10.7759/cureus.39536

**Published:** 2023-05-26

**Authors:** Nazima Khatun, Aboud Kaliounji, Sami S Alkoutami, Jonathan Francois, Sabu John

**Affiliations:** 1 Internal Medicine, State University of New York (SUNY) Downstate Medical Center, Brooklyn, USA; 2 Internal Medicine, St. George's University School of Medicine, St. George's, USA; 3 Cardiology, State University of New York (SUNY) Downstate Medical Center, Brooklyn, USA; 4 Cardiology, Kings County Hospital Center, Brooklyn, USA

**Keywords:** aortic valve insufficiency, echocardiography, adult congenital heart disease, aortic valve, quadricuspid aortic valve

## Abstract

Quadricuspid aortic valve (QAV) is a very rare congenital abnormality. Here, we present a rare case of QAV incidentally noted in a patient at an advanced age during transthoracic echocardiography (TTE). A 73-year-old man with a history of hypertension, hyperlipidemia, diabetes, and treated prostate cancer was admitted to the hospital with palpitations. An electrocardiogram (ECG) showed T-wave inversion in V5-V6, with initial troponin levels mildly elevated. Acute coronary syndrome was ruled out by serial ECGs that were unchanged, and troponins downtrended. TTE showed a rare and incidental finding of type A QAV with four equal cusps with mild aortic regurgitation.

## Introduction

Quadricuspid aortic valve (QAV) is a rare congenital abnormality that was first reported in 1847 by Dr. Benjamin Guy Babington in his original case report describing the autopsy findings in a cyanotic 34-year-old woman with patent ductus arteriosus and a four-leaflet aortic valve [1]. A normal aortic valve has three cusps, but cases of bicuspid, unicuspid and quadricuspid aortic valve, which is rare, have been reported. The incidence of QAVs in echocardiography is 0.0059%-0.0065%, although the rate remains underestimated and varies among publications [2].

Transthoracic echocardiography (TTE) is most commonly used for diagnosis; however, diagnosis can also be made by surgery, aortography, autopsy, and other imaging modalities such as transesophageal echocardiography (TEE) and cardiac magnetic resonance imaging (MRI) [3]. While most patients with QAV are asymptomatic, progression to valvular disease, particularly aortic insufficiency, can be seen depending on the relative cusp sizes. While not all patients with QAV require surgical repair, limited data exist on the long-term survival benefits of surgical correction [2]. Here, we present a case of an incidental finding of QAV in an African American 73-year-old male.

## Case presentation

An African American 73-year-old man, who recently moved to the United States, with a past medical history of hypertension, hyperlipidemia, type II diabetes mellitus, and prostate cancer status post-resection presented to the emergency department for palpitations. The patient reported that the palpitations had been present for the past year but had increased in the last three days. The patient described the palpitations as episodic, lasting a few seconds to minutes, and worse at night. He denied any association with movement or exertion and reported to maintain good exercise tolerance of 10 city blocks. The patient denied chest pain, shortness of breath, nausea, vomiting, headaches, or dizziness during these episodes.

His vital signs were as follows: blood pressure 193/65 mmHg, pulse 76 beats per minute (bpm), and temperature 97.9°F. Physical examination was unremarkable. Initial electrocardiogram (ECG) in the emergency room showed a T-wave inversion in V5-V6; no prior ECG was available to compare. Preliminary labs showed a glucose level of 120 mg/dL, blood urea nitrogen 60 mg/dL, creatinine 3.62 mg/dL, and eGFR 17 mL/min/1.73 m^2^. The pro-brain natriuretic peptide level was 881 pg/mL and troponins were at 0.053 and 0.048 ng/mL. The patient was anemic with hemoglobin at 8.9 g/dL and a hematocrit of 27.5% with an unknown baseline. Preliminary labs are summarized in Table 1. His chest x-ray was unremarkable. The patient was given carvedilol, hydralazine, and isosorbide dinitrate for his hypertension; his blood pressure subsequently reduced to 147/90 mmHg and stabilized at 118/72 mmHg. The patient was then admitted to the medicine floor for the evaluation of palpitations, to rule out acute coronary syndrome (ACS) and management of uncontrolled hypertension.

**Table 1:** Laboratory results on admission

Tests	Results	Reference ranges
Troponin T	0.053	<0.04 ng/mL
Pro-brain natriuretic peptide	881	<100 pg/mL
Blood urea nitrogen	60	7-20 mg/dL
Creatinine	3.62	0.6-1.1 mg/dL
Estimated glomerular filtration rate	17	≥60 mL/min/1.73 m^2^
Hemoglobin	8.9	11.2-15.7 g/dL
Hematocrit	27.5	34%-45%
Glucose	120	77-100 mg/dL

**Figure 1:**
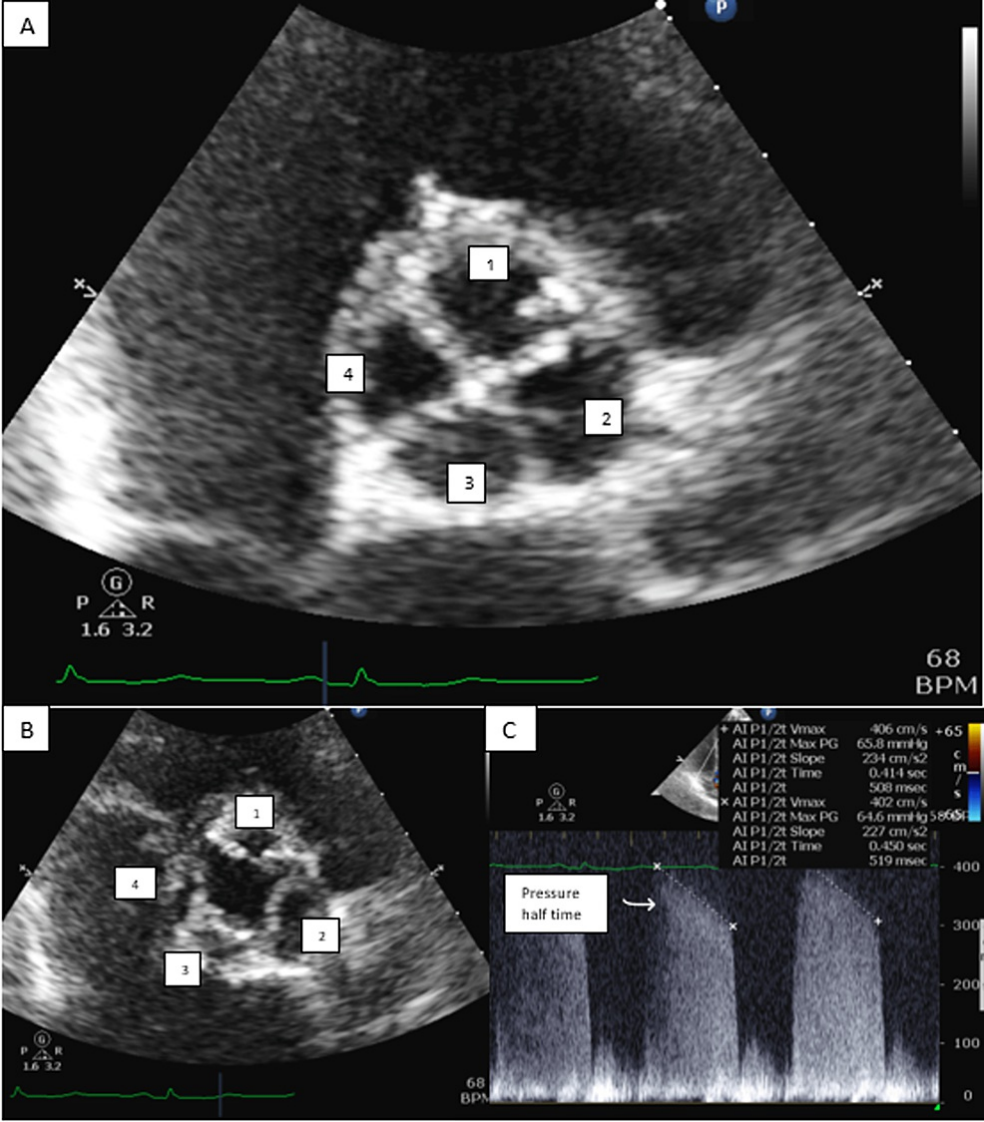
TTE images TTE (parasternal short-axis view) shows four equal cusps of the aortic valve in (A) diastole and (B) systole. (C) Mild aortic regurgitation by pressure half-time value of regurgitant jet greater than 500 msec. TTE, transthoracic echocardiography

Serial ECGs were unchanged, and the troponin level downtrended to 0.010 ng/mL. Telemetry monitoring showed no arrhythmias. An echocardiogram was ordered and showed a left ventricle ejection fraction between 55% and 60% and an incidental finding of a quadricuspid aortic valve as shown in Figure 1 (panels A and B), with mild regurgitation but no stenosis (panel C).

The cardiology department was consulted for his ECG results but did not recommend any acute intervention for QAV as only mild AR was noted on TTE; a follow-up in the outpatient clinic was recommended for further workup for his palpitations including Holter monitoring. During the two-day admission, the patient was given carvedilol, isosorbide dinitrate, hydralazine, atorvastatin, heparin, insulin, and tamsulosin. After two days in the hospital, the patient was discharged as his blood pressure stabilized at 122/78 mmHg and his palpitations resolved. His discharge medication included carvedilol 6.25 mg twice daily along with hydrochlorothiazide 12.5 mg daily, atorvastatin 20 mg daily, tamsulosin 0.4 mg daily, and insulin glargine. Upon discharge, the patient was instructed to follow up with the cardiology department.

Two weeks after discharge, the patient returned to the cardiology clinic for continued palpitations. The patient denied any symptoms of shortness of breath, chest pain, or a decrease in exercise tolerance. The Holter monitor showed that the patient had a minimum heart rate of 59 bpm, maximum heart rate of 169 bpm, and an average heart rate of 75 bpm. The predominant underlying rhythm was sinus rhythm, and 13 supraventricular tachycardia (SVT) runs occurred with the longest duration being 15 beats. These runs of SVTs were not associated with the patient’s symptoms. Due to the palpitations, the carvedilol dose was increased from 6.25 mg twice daily to 12.5 mg twice daily. His blood pressure in the clinic was 190/73 mmHg, and nifedipine 30 mg daily was added to help control his blood pressure. On the next follow-up visit, the patient reported that his palpitations resolved and his blood pressure in the clinic was 161/71 mmHg. The nifedipine dose was further increased to 60 mg daily.

## Discussion

Because of congenital anomalies, an increase in the number of cases of bicuspid, unicuspid and quadricuspid aortic valve, which is rare, has been reported. With advanced technology and imaging modalities over the past decades, such as cardiac magnetic resonance imaging, transthoracic echocardiography, and transesophageal echocardiography, the prevalence of QAV in asymptomatic individuals has been found to be increased. In their study at the Mayo Clinic of 788,733 patients who got an echocardiogram between 1975 and 2014, Tsang et al. found that 52% of the patients with QAV were females [2]. They also found that the mean age at diagnosis was 43.5 years. While the true mechanism of this valvular disease remains unknown, it is believed to be due to an inflammatory process leading to abnormal septation of endocardial cushions and anomalous septation of the conotruncus [4]. Although QAV is usually an isolated anomaly, another associated cardiac defect can be present with coronary ostium, coronary artery anomalies being the most common [5]. Other common anomalies include mitral valve malformation, pulmonary stenosis, ventricular septal defects and patent ductus arteriosus [4].

Hurwitz and Roberts identified seven anatomical subtypes of QAV lettered A to G based on relative cusp sizes [6]. In type A, there are four equal-sized cusps; in type B, one smaller cusp and three equal-sized cusps; in type C, two equal smaller cusps and two equal larger cusps; in type D, one bigger cusp, one smaller cusp, and two medium-sized cusps; in type E, one larger cusp and three equal-sized cusps; in type F, two same-sized cusps and two smaller cusps with different sizes and in type G, four unequal-sized cusps.

QAVs are clinically silent in most young patients, but older individuals are prone to develop aortic insufficiency due to progressive leaflet fibrosis and failure of leaflet coaptation. In a study, Tutarel and Westhoff-Bleck found that aortic regurgitation (AR) was seen in 74.7% cases of QAV, stenosis alone in 0.7%, stenosis and regurgitation in 8.4% and intact valve in 16.2% cases [7]. There are no recent studies evaluating which subtypes have a higher risk of developing AR. It is in fact still unknown whether the classification of different subtypes has distinct prognostic implications. One can hypothesize that the presence of unequal cusp sizes can lead to unequal distribution of stress, leading to leaflet fibrosis, which eventually increases the risk of AR. On the other hand, QAVs with four equal cusps are less likely to develop fibrous thickening due to the equal distribution of force on the valve.

Clinical presentation depends on the morphology and functionality of the valve that leads to the presence of other associated disorders. Shortness of breath, fatigue, syncope, pedal edema, chest pain, and palpitations have been reported in QAV patients [8]. Since most QAV patients are found to have AR, the structural changes in the valve and leaflet fibrosis can increase the susceptibility of patients to other complications including endocarditis and rheumatic valve disease. In addition, only a few cases of congestive heart failure have been reported. Salum et al. described a case of a 56-year-old female with diagnosed QAV who presented with progressive AR and severe heart failure [9]. Tsujimoto et al. described another case of idiopathic dilated cardiomyopathy in a 47-year-old male with QAV [10]. The purpose of this case is to highlight the possible late complications including palpitations and congestive heart failure in patients with congenital QAV. It is also imperative to note the importance of diagnostic screening in third-world countries and the need to increase healthcare access in those areas; our patient was diagnosed with QAV at 73 while the mean age is 43.5 years.

Since the majority of QAVs are asymptomatic, close monitoring is required to assess the progression of the disease given the high prevalence of AR, especially in the younger patients who are more likely to undergo surgery later in life. Although there are no guidelines regarding the surveillance of patients with QAV, physicians should discuss the need for surveillance with TTE depending on the patient’s comorbidities and the risk of developing progressive hemodynamic concessions. Although there remains an absence of evidence-based standards for surgical repair, patients with malfunctioning QAV, severe aortic stenosis, or regurgitation, corresponding to one-fifth of patients, are usually referred for surgical repair, which is the standard treatment [3]. In another study, 45.2% of the 186 QAV cases were referred to surgery during the fifth to sixth decade of life [7]. The mean duration between the time of diagnosis and surgery was 4.3 years [2]. In fact, there was no significant difference in the overall mortality in surgical versus non-surgical patients [2]. Tricuspidalization, which is the favored aortic valve repair technique, is recommended over aortic valve replacement given a lower risk of bleeding, infective endocarditis, prosthetic valve degeneration, and thromboembolism compared to valve repair [11]. Because of its low incidence and rarity, the long-term effects, complications, and disease progression are poorly understood and require further investigation.

## Conclusions

QAV is a rare congenital heart disease with a prevalence of less than 0.05%. QAV can be associated with aortic valve dysfunction and aortic root dilatation. In this case, QAV was an incidental finding that was associated with only mild AR at an advanced age. This case also highlights the importance of TTE in diagnosing QAV.
